# A human serum albumin-assisted caged fluorophore probe for fluorescence turn-on detection of hypochlorous acid

**DOI:** 10.1039/d6ra06489b

**Published:** 2026-09-18

**Authors:** Hyun Seo Kim, Min Su Han

**Affiliations:** a Department of Chemistry, Gwangju Institute of Science and Technology (GIST) 123 Cheomdangwagi-ro, Buk-gu Gwangju 61005 Republic of Korea happyhan@gist.ac.kr

## Abstract

Fluorescence detection of hypochlorous acid (HOCl), a reactive oxidant that modulates immune responses and inflammation, can be complicated in serum due to nonspecific interactions between fluorescent probes and serum proteins. In particular, human serum albumin (HSA), the most abundant protein in serum, can alter the probe fluorescence by increasing background signals and distorting analyte-dependent responses. Rather than suppressing this protein-induced effect, we sought to convert albumin binding into a controlled signal-generation step. Here, we report DSH, an HSA-assisted caged fluorophore probe for HOCl detection in albumin-containing media. DSH was constructed by masking dansyl-sarcosine (DS) with a 2,4-dinitrophenyl caging group through a hydrazine-based linker that undergoes oxidative cleavage in response to HOCl. In the caged state, fluorescence is suppressed, and direct albumin-induced activation is minimised. Upon exposure to HOCl, oxidative cleavage of the linker releases DS, which subsequently binds to HSA and produces a fluorescence turn-on response. Thus, HSA binding, typically considered a source of matrix interference, is repurposed as a signal-amplifying step in the sensing mechanism. This DSH-based sensing strategy enables calibration-based quantitative detection of HOCl in phosphate-buffered saline supplemented with HSA and diluted human serum, supporting the feasibility of this caged-fluorophore design under controlled albumin-containing conditions.

## Abstract 2

Fluorescence detection of hypochlorous acid (HOCl) in serum can be complicated by nonspecific probe–protein interactions. To address this issue, we designed an HSA-assisted caged fluorophore probe for HOCl detection.

## Graphical abstract

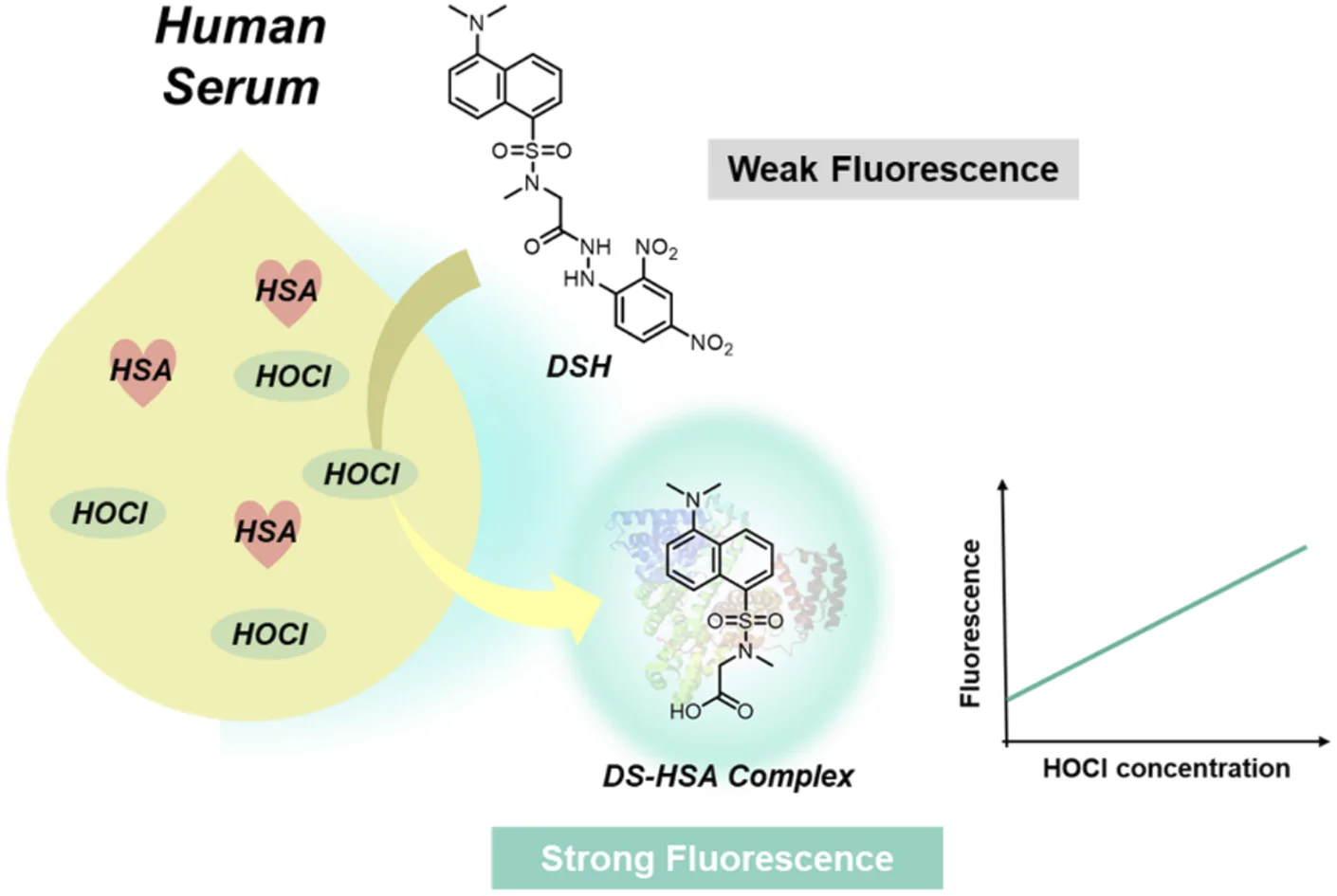

## Introduction

Hypochlorous acid (HOCl) is a reactive oxidant produced by myeloperoxidase (MPO) in activated neutrophils and macrophages, in the presence of hydrogen peroxide (H_2_O_2_) and chloride ions (Cl^−^) as substrates.^1^ Under physiological conditions, HOCl contributes to innate immune defence by reacting with invading pathogens.^2–4^ However, excessive or dysregulated HOCl can diffuse into surrounding tissues and oxidise various biomolecules, leading to cellular damage and tissue injury.^5,6^ Such oxidative damage has been associated with inflammatory and degenerative diseases including hepatic ischemia-reperfusion injury, atherosclerosis, lung injury, and rheumatoid arthritis.^7,8^ Hence, reliable detection and monitoring of HOCl in biological systems are important for understanding its pathological roles and evaluating oxidative stress-related disease states.^9,10^

Fluorescence-based sensing has emerged as an attractive approach for HOCl detection owing to its high sensitivity, operational simplicity, and excellent spatiotemporal resolution.^11–15^ However, reliable fluorescence detection in human serum remains challenging because of the presence of abundant proteins and other biomolecules that can interact non-specifically with probes and distort analyte-dependent fluorescence signals.^16,17^ In particular, human serum albumin (HSA), the most abundant serum protein, can bind to small-molecule fluorescent probes and alter their photophysical properties.^18–21^ Such HSA-probe interactions can increase background fluorescence and mask the target response, leading to inaccurate quantitative measurements in serum.^17^

Beyond efforts to minimise protein-induced interference, several studies have explored protein-assisted fluorescence signal generation rather than treating protein interactions solely as sources of interference.^21^ In particular, serum albumin and endogenous cellular proteins have been utilised to enhance fluorescence signals through protein-fluorophore interactions, including albumin-assisted HOCl sensing and cytoplasmic protein-powered *in situ* fluorescence amplification.^22,23^ HSA has also been exploited as an intrinsic signal amplifier for biomarker detection in human plasma.^24^

Our group has also applied this concept to target-responsive fluorescence sensing using dansyl-sarcosine (DS).^25,26^DS is an albumin-binding fluorophore that exhibits strong fluorescence upon binding to the hydrophobic pocket of HSA, particularly Sudlow's site II.^27^ In this approach, DS is masked by a quencher through an analyte-cleavable linker in order to suppress background fluorescence and prevent direct albumin-induced activation before analyte recognition. After reaction with the target analyte, cleavage of the linker releases DS, which binds to HSA and generates a fluorescence turn-on signal. Thus, instead of treating albumin binding as an interference, this platform uses HSA binding as the essential part of signal generation.

**Fig. 1:**
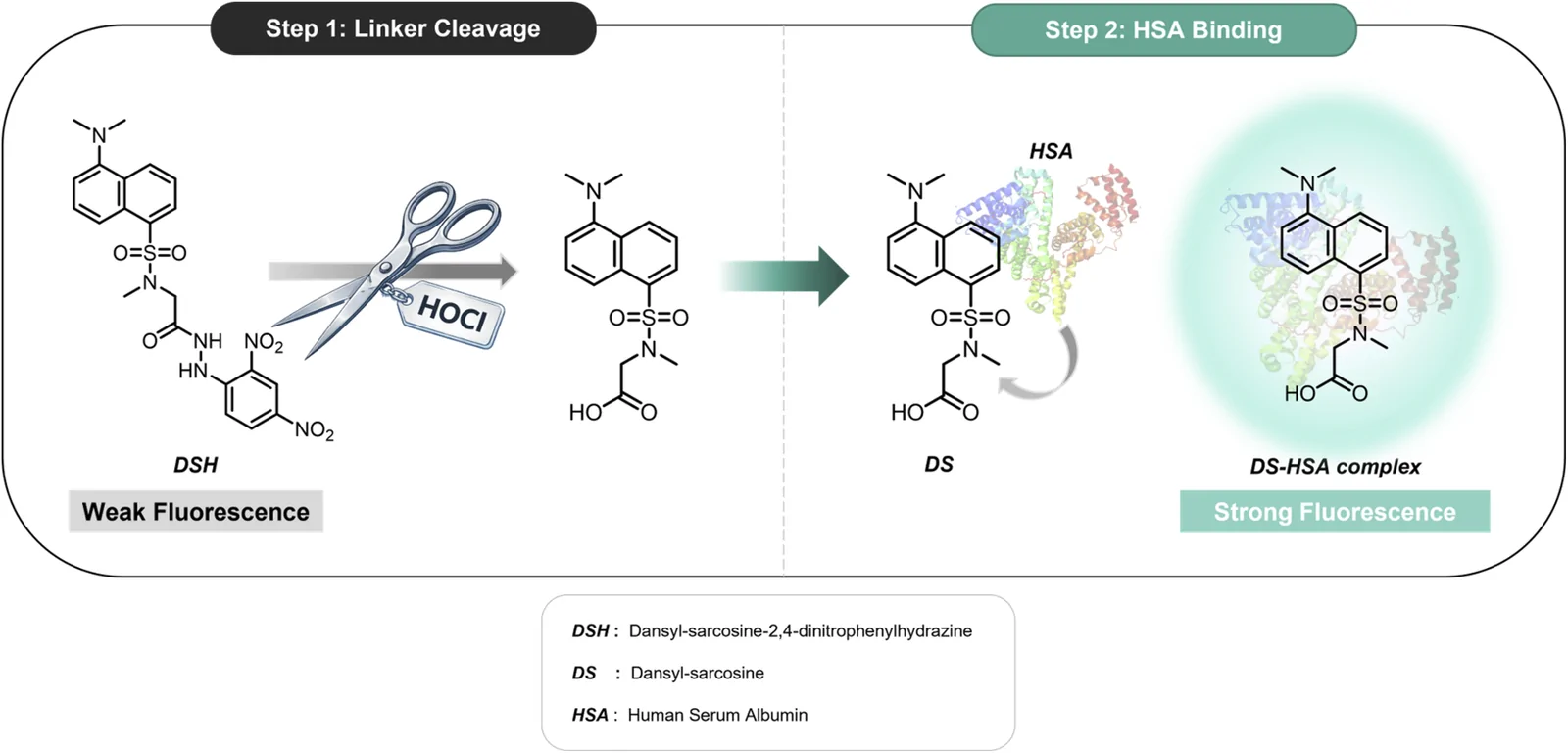
Schematic illustration of the HOCl-triggered albumin-assisted fluorescence turn-on response of DSH.

Building on this strategy, we developed DSH as an HOCl-responsive caged fluorophore for albumin-assisted fluorescence detection (Fig. 1). DSH was constructed by caging the albumin-binding fluorophore DS with a 2,4-dinitrophenyl group through an HOCl-cleavable hydrazine-based linker. This molecular design aims to suppress the intrinsic fluorescence of DS and prevent direct HSA-induced fluorescence activation before reaction with HOCl. Upon exposure to HOCl, oxidative cleavage of the linker was expected to release DS and enable subsequent HSA binding, thereby generating a strong fluorescence turn-on response. Based on this design, we evaluated the HOCl-responsive behaviour of DSH and demonstrated the calibration-based fluorescence turn-on detection of HOCl in HSA-containing buffers and diluted human serum, thereby supporting the use of albumin binding as a signal-generating component of the caged-fluorophore platform.

## Experimental

### Materials and instruments

All chemical reagents were purchased from commercial suppliers (Tokyo Chemical Industry, Sigma-Aldrich, Alfa Aesar, DUKSAN E&P and DAEJUNG) and used without further purification. Human serum (H6914) was commercially purchased from Sigma-Aldrich and used for the serum-containing assays.

^1^H NMR (400 MHz) and ^13^C NMR (101 MHz) spectra were recorded on a JEOL (400 MHz) NMR spectrometer. UV-vis spectra were recorded using a JASCO V-630 UV-vis spectrometer, and fluorescence spectra were measured using an Agilent Cary Eclipse fluorescence spectrometer. High-resolution mass spectra (HRMS) were recorded on a Bruker Impact II QTof mass spectrometer with an electrospray ionisation source. Liquid chromatography-triple quadrupole mass spectrometry (LC-TQMS) analyses were performed using a Bruker EVOQ Elite system equipped with a heated electrospray ionisation source. Chromatographic separation was performed on a Waters BEH C18 column (2.1 × 150 mm, 1.7 µm).

### Synthesis of DS and DSH

DS was synthesised according to a literature procedure and used as the albumin-binding fluorophore precursor (see the SI for details). DSH was synthesised according to a modified literature procedure.^28^DS (161 mg, 0.50 mmol) was dissolved in DMF (5 mL), and 1-[bis(dimethylamino)methylene]-1*H*-1,2,3-triazolo[4,5-*b*]pyridinium 3-oxide hexafluorophosphate (HATU; 228 mg, 0.60 mmol) was added. *N*,*N*-Diisopropylethylamine (DIEA; 104.5 µL, 0.60 mmol) was then added, and the reaction mixture was stirred at room temperature for 1 h. Subsequently, 2,4-dinitrophenylhydrazine (149 mg, 0.75 mmol) was added, followed by the addition of DIEA (174.2 µL, 1.00 mmol). The reaction mixture was stirred at room temperature for an additional 5 h. The reaction progress was monitored by TLC using hexane/ethyl acetate (1 : 1, v/v), which revealed the appearance of a new spot (*R*_f_ = 0.32). After completion of the reaction, the reaction mixture was diluted with dichloromethane and washed with water and brine. The organic layer was dried over anhydrous Na_2_SO_4_, filtered, and concentrated under reduced pressure. The crude product was purified by silica gel column chromatography using hexane/ethyl acetate (1 : 1, v/v) as the eluent to afford DSH as a brown solid in 65% yield. ^1^H NMR (400 MHz, CDCl_3_ with TMS) *δ* 9.46 (s, 1H), 9.17 (s, 1H), 8.87 (d, *J* = 2.3 Hz, 1H), 8.56 (d, *J* = 8.7 Hz, 1H), 8.26–8.07 (m, 3H), 7.51 (t, *J* = 8.0 Hz, 2H), 7.16–7.05 (m, 2H), 3.99 (s, 2H), 3.03 (s, 3H), 2.88 (s, 6H). ^13^C NMR (101 MHz, CDCl_3_ with TMS) *δ* 168.0, 152.3, 148.2, 138.4, 131.9, 131.7, 131.0, 130.6, 130.2, 130.0, 128.9, 123.3, 123.2, 118.4, 115.6, 114.8, 52.7, 45.4, 37.2. HRMS (ESI): *m*/*z* calculated for [M + H]^+^ 503.1364, found 503.1361.

### General fluorescence measurements

All sample solutions (1.0 mL) were prepared in 1.5 mL Eppendorf tubes. All fluorescence measurements were performed in triplicate at room temperature. Unless otherwise stated, DSH (10 µM) was added to 1× phosphate-buffered saline (PBS, pH 7.4) containing HSA (20 µM), HOCl at the indicated concentration, and 1% DMSO. The sample solutions were incubated for 90 min at room temperature before fluorescence measurement. *λ*_ex_ = 340 nm. The fluorescence intensity at *λ*_em_ = 485 nm was used for data analysis. Slit widths: 10/10 nm unless otherwise noted. PMT voltage: 600 V.

### Selectivity and interference studies

Selectivity experiments were performed under the general fluorescence conditions described above. HOCl was used at a final concentration of 100 µM, whereas other species were used at 2 mM concentration. According to the literature, ROO˙, NO˙, and ˙OH were generated from 2,2′-azobis(2-amidinopropane) dihydrochloride (AAPH), sodium nitroprusside (SNP), and a Fenton-type reaction, respectively.^29^ Glutathione (GSH) and cysteine (Cys) were directly added from aqueous stock solutions prepared in TDW.

For the metal interference studies, the HOCl concentration was fixed at 100 µM, and each metal salt was added under the same assay conditions. The metal salts and final concentrations were as follows: KCl (10 mM), MgCl_2_ (10 mM), CaCl_2_ (2 mM), Fe(ClO_4_)_3_ (100 µM), FeCl_2_·_4_H_2_O (100 µM), CuCl_2_ (20 µM), ZnCl_2_ (100 µM), MnCl_2_ (100 µM), and HgCl_2_ (100 µM).

### Detection of HOCl in diluted human serum

For serum-containing assays, HSA was replaced with diluted human serum under otherwise identical fluorescence conditions. Commercial human serum (Sigma-Aldrich) was diluted with 1× PBS (pH 7.4) to prepare 1%, 2%, 5%, and 10% serum-containing assay conditions. For serum matrix evaluation, HOCl was added at final concentrations of 0, 50, and 100 µM, corresponding to 0, 5, and 10 equivalents relative to DSH. For calibration-based detection, different concentrations of HOCl were added to 3% and 10% human serum-containing PBS solution. The final DMSO content was maintained at 1%.

For spike-recovery analysis, HOCl was added to 3% and 10% human serum at final concentrations of 10 and 50 µM. Found HOCl concentrations were calculated using matrix-matched calibration curves constructed over the 0–10 equiv. HOCl range under the corresponding serum condition. Recovery and relative standard deviation (RSD) values were calculated from triplicate measurements.

### Mechanistic validation by LC-TQMS

The HOCl-mediated conversion of DSH to DS was examined by LC-TQMS analysis. DSH was incubated with HOCl in PBS buffer (1×, pH 7.4) containing 1% MeCN at room temperature. HOCl was added at 10 or 20 equiv. relative to DSH, and the reaction mixtures were incubated for 1 h. HOCl-free DSH and DS controls were prepared under otherwise identical conditions. After incubation, residual HOCl in the HOCl-treated samples was quenched with methionine, and all samples were desalted prior to LC-TQMS analysis. The resulting samples were analysed by reversed-phase LC-TQMS. DSH and DS were monitored in positive-ion selected ion monitoring (SIM) mode at *m*/*z* 503.2 and 323.2, respectively, using predefined retention-time windows. The LC-TQMS conditions were as follows: 2% B for 0.5 min; 2–100% B over 9.5 min; 100% B for 3 min; 100–2% B over 0.1 min; 2% B for 1.9 min. Mobile phase A: water containing 0.1% formic acid; mobile phase B: acetonitrile containing 0.1% formic acid. The flow rate was 0.3 mL min^−1^. The MS conditions were as follows: heated electrospray ionisation (HESI) source; positive mode; spray voltage, 3500 V in the positive mode; cone temperature, 300 °C; probe temperature, 275 °C.

## Results and discussion

### Design and photophysical properties of DSH

DSH was designed as an HOCl-responsive caged fluorophore based on an albumin-assisted signal-generation strategy. As illustrated in Fig. 1, the sensing mechanism involves two sequential steps: HOCl-induced oxidative cleavage of the linker to release DS, followed by the binding of DS to HSA to form a fluorescent complex. In the intact probe, DS is masked with a 2,4-dinitrophenyl group through a hydrazine-based linker. The 2,4-dinitrophenyl group was selected as a caging and quenching unit because it can suppress the fluorescence of the dansyl fluorophore, while hydrazine or hydrazone-type motifs undergo oxidative cleavage in response to HOCl.^6,7,30–32^ Therefore, DSH was expected to be weakly fluorescent before reaction with HOCl. DS was synthesised according to a reported procedure, and DSH was subsequently prepared from DS and the 2,4-dinitrophenylhydrazine-based caging unit. Before evaluating DSH, the HSA-dependent fluorescence behaviour of DS was examined as a control. DS exhibited strong fluorescence only in the presence of HSA, whereas DS alone and DS treated with HOCl showed weak emission under the same assay medium (Fig. S3). Moreover, the HSA-induced fluorescence of DS was also largely independent of HOCl, as similar emission intensities were observed in the presence of HSA with or without HOCl. This result confirmed the role of DS as a signal-generating fluorophore after DSH de-caging.

**Fig. 2:**
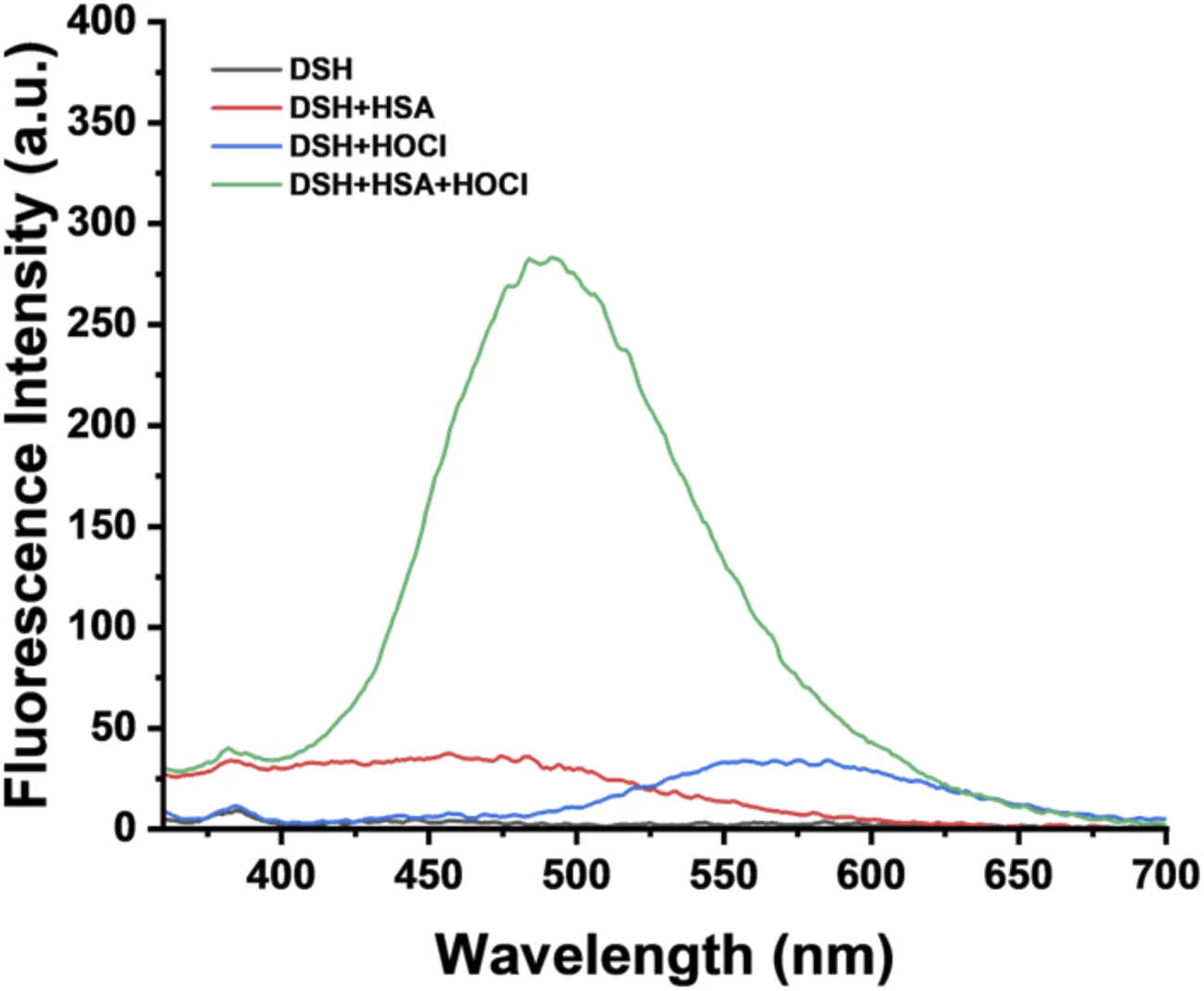
Fluorescence emission spectra of DSH in the absence and presence of HSA, HOCl, or both. DSH = 10 µM, HSA = 20 µM, HOCl = 100 µM, *λ*_ex_ = 340 nm, and measurements were performed in 1× PBS (pH 7.4) containing 1% DMSO after incubation for 90 min at room temperature.

The photophysical behaviour of DSH was then examined under different combinations of HSA and HOCl. As shown in Fig. 2, DSH alone showed negligible fluorescence upon excitation at 340 nm, indicating that the fluorescence of DS was effectively suppressed in the intact probe. The addition of either HOCl or HSA alone resulted in a slight fluorescence enhancement. In contrast, a pronounced fluorescence turn-on response was observed when both HOCl and HSA were present. These results indicated that the direct albumin-induced activation of DSH is largely suppressed before reaction with HOCl. On the other hand, HOCl-mediated probe activation followed by HSA binding produced a strong fluorescence signal.

**Fig. 3:**
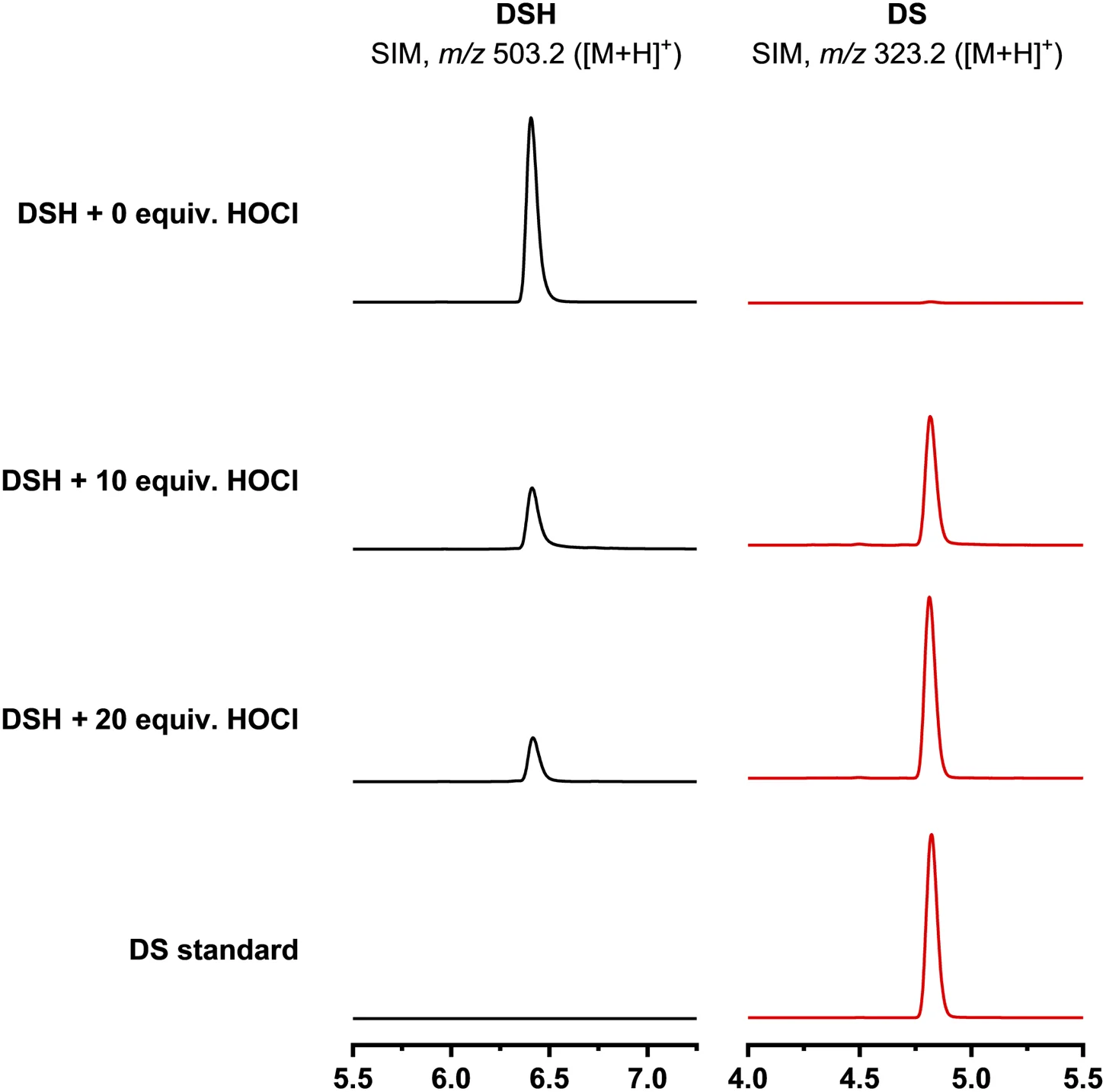
LC-TQMS SIM chromatograms of an HOCl-free DSH control, DSH reaction mixtures treated with 10 or 20 equiv. HOCl, and an HOCl-free DS standard in 1× PBS (pH 7.4), supporting the HOCl-mediated de-caging of DSH to DS.

To support the proposed HOCl-mediated de-caging process, the reaction mixture was analysed by LC-TQMS in SIM mode. As shown in Fig. 3, the DSH-targeted signal decreased following treatment with 10 and 20 equiv. HOCl, while a signal corresponding to DS was observed at the retention time of the HOCl-free DS control. These results support the HOCl-mediated conversion of the caged probe DSH to the uncaged fluorophore DS under the PBS condition used for the fluorescence measurements.

### Optimisation for quantitative HSA-assisted HOCl detection

To establish suitable conditions for calibration-based HOCl detection, the effects of the buffer and pH were examined (Fig. S4). DSH showed a clear HOCl-dependent fluorescence response in PBS and phosphate buffer. However, the response was strongly attenuated in Tris buffer, likely due to the consumption of HOCl by the amine-containing buffer component.^33^ The fluorescence response was stronger under mildly acidic conditions, likely because the HOCl/ClO^−^ equilibrium shifted toward the more reactive HOCl species at lower pH.^34^ Based on these results, 1× PBS at pH 7.4 was selected as the assay medium to maintain near-physiological conditions. The HSA concentration was then optimised under the selected PBS condition. The fluorescence response of DSH was measured in the presence of HOCl while varying the HSA concentration from 0 to 25 µM, corresponding to 0–2.5 equiv. relative to the probe. As shown in Fig. S5, the fluorescence intensity increased with increasing HSA concentration. The spectra obtained with 20 and 25 µM HSA showed nearly identical fluorescence intensities, indicating that the fluorescence response was approaching saturation under these conditions. Therefore, 20 µM HSA, corresponding to 2 equiv. relative to DSH, was selected as the optimised HSA concentration for subsequent fluorescence measurements.

Time-dependent fluorescence measurements were performed to examine the response behaviour of DSH toward HOCl under the HSA-assisted conditions (Fig. S6a). The fluorescence intensity gradually increased after HOCl addition, with higher HOCl equivalents giving stronger responses. Clear concentration-dependent signal separation was observed within the first 30 min, so that the apparent initial fluorescence increase rates at various HOCl concentrations could be compared (Fig. S6b). To further evaluate the early fluorescence response of DSH toward HOCl, a calibration plot was constructed using the fluorescence intensities measured at 10 min after HOCl addition. A clear concentration-dependent response was observed over the tested HOCl concentration range (Fig. S7). The corresponding detection limit was calculated to be 2.21 µM. Although the HOCl-dependent response was already distinguishable at this early time point, the fluorescence intensity continued to increase with time, providing a larger signal window at later time points. Accordingly, 90 min was retained as a standardised endpoint for subsequent quantitative measurements to provide a larger signal window and improved analytical sensitivity under consistent assay conditions.

### Detection limit determination for DSH-based HSA-assisted HOCl sensing

**Fig. 4:**
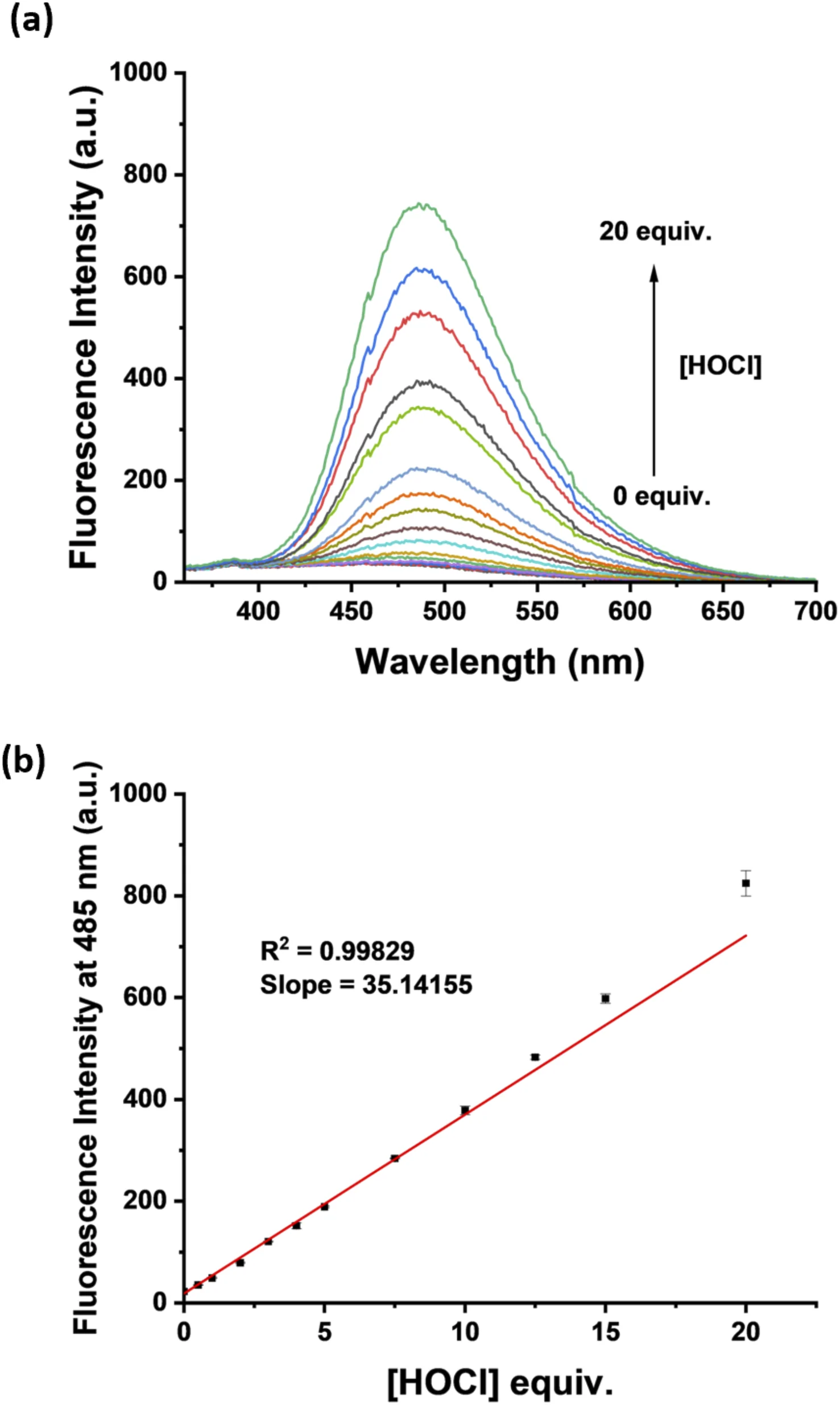
Quantitative fluorescence response of DSH toward HOCl under HSA-assisted sensing conditions. (a) Fluorescence emission spectra of DSH recorded with increasing concentrations of HOCl from 0 to 20 equiv. relative to DSH. (b) Calibration plot of fluorescence intensity at 485 nm *versus* HOCl equiv. measurements were performed under the general fluorescence conditions using HSA (20 µM), except that the HOCl concentration was varied.

Under the optimised conditions, the fluorescence response of DSH was investigated by increasing the HOCl concentration from 0 to 20 equiv. relative to DSH in PBS/HSA. As shown in Fig. 4a, the emission intensity at 485 nm gradually increased with increasing HOCl concentration, indicating HOCl-dependent fluorescence turn-on behaviour. The corresponding calibration plot showed a linear relationship between the fluorescence intensity and the HOCl concentration over the tested quantitative range (Fig. 4b). The detection limit was calculated to be 0.66 µM using the equation LOD = 3*σ*/*k*, where *σ* is the standard deviation of the blank signal and *k* is the slope of the calibration curve. This result demonstrated micromolar-level albumin-assisted fluorescence detection of HOCl under the defined PBS/HSA conditions.

A comparison with representative fluorescent probes for HOCl detection is provided in Table S1, highlighting the distinct HSA-assisted sensing strategy of DSH.

### Selectivity and interference studies

**Fig. 5:**
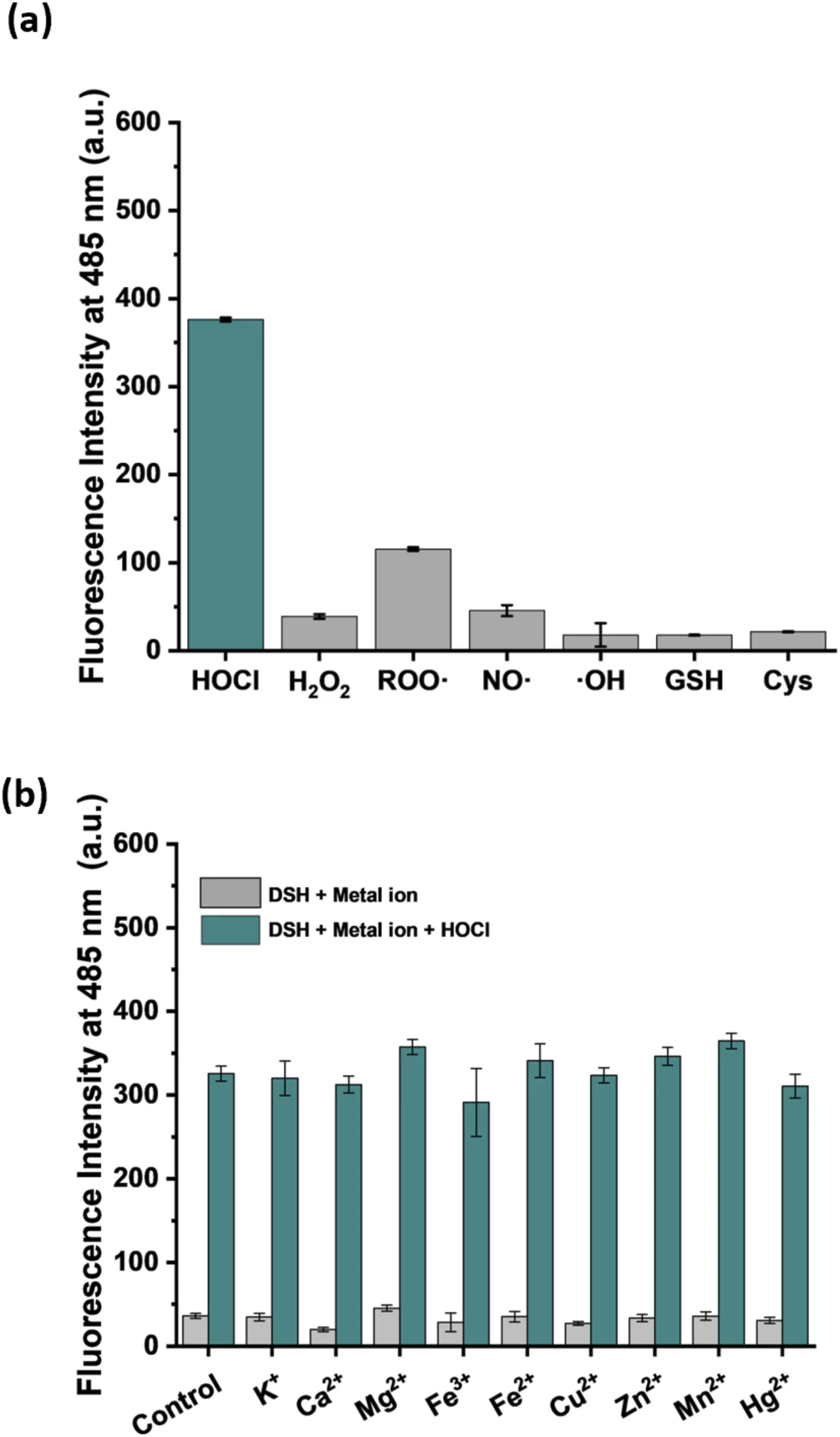
Selectivity and metal-ion interference studies of DSH under HSA-assisted sensing conditions. (a) Fluorescence response of DSH toward HOCl, other reactive oxygen and nitrogen species, and biologically relevant thiols. (b) Fluorescence response of DSH in the presence of metal ions with or without HOCl. Fluorescence intensities were recorded at 485 nm under the general fluorescence conditions using HSA (20 µM). For the selectivity study in panel (a), HOCl was used at 100 µM, whereas the other tested ROS/RNS and biothiols were used at 2 mM.

The selectivity of the sensing system was evaluated against other reactive oxygen and nitrogen species as well as biologically relevant thiols. To evaluate the selectivity of DSH under excess interferent conditions, H_2_O_2_, ROO˙, NO˙, ˙OH, GSH, and Cys were tested at 2 mM, corresponding to 20-fold excess relative to HOCl. As shown in Fig. 5a, HOCl led to the strongest fluorescence enhancement among the tested species, indicating that the fluorescence turn-on response of DSH in the presence of HSA is preferentially triggered by HOCl over the tested ROS/RNS and biothiols.

The potential interference of metal ions was also examined, because metal ions in biological samples can affect fluorescence measurements through binding, quenching, or redox-related processes. The tested ions included abundant biological cations (K^+^, Mg^2+^, and Ca^2+^), redox-active transition metal ions (Fe^3+^, Fe^2+^, Cu^2+^, and Mn^2+^), and ions that may interact with fluorescent ligands or proteins (Zn^2+^ and Hg^2+^). The corresponding salt forms and final concentrations are mentioned in the Experimental section. These concentrations were selected to represent physiologically relevant or excess interferent conditions. As shown in Fig. 5b, fluorescence intensity remained low in the absence of HOCl, even in the presence of metal ions. Upon addition of HOCl, fluorescence enhancement was sustained under all tested metal ion conditions. These results indicated that the HOCl-dependent, albumin-assisted response of DSH is largely preserved in the presence of the tested metal interferents.

### Fluorescence response in a cell-free MPO/H_2_O_2_ system

To further examine the response of DSH under an enzymatic condition expected to generate HOCl, a cell-free MPO/H_2_O_2_ system was tested in PBS/HSA. Measurements were performed according to the general fluorescence procedure described above, using DSH (10 µM), HSA (20 µM), MPO (0.07 U mL^−1^), and H_2_O_2_ (10 µM) in 1× PBS (pH 7.4) containing 1% DMSO, with incubation for 90 min at room temperature. Control samples were prepared by omitting either MPO, H_2_O_2_ or both from otherwise identical mixtures. Only minor fluorescence changes were observed in the absence of either MPO or H_2_O_2_, whereas the complete MPO/H_2_O_2_ system showed a modest increase in fluorescence (Fig. S8). These observations suggest that DSH can exhibit a measurable fluorescence response under the tested MPO/H_2_O_2_ condition.

### Detection of HOCl in diluted human serum

**Fig. 6:**
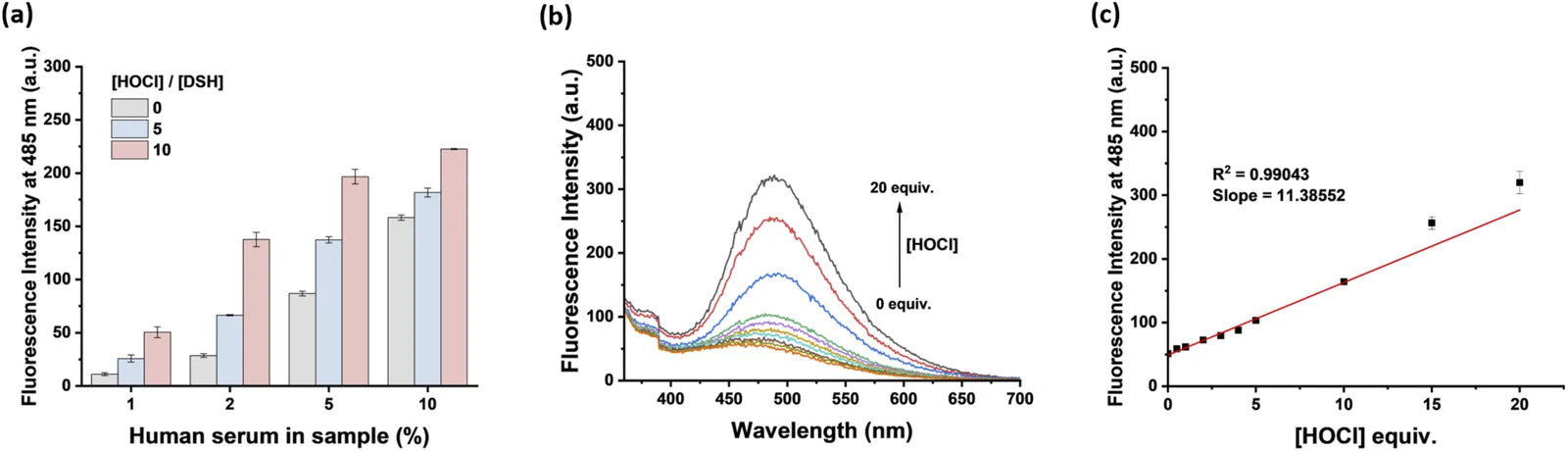
Detection of HOCl in diluted human serum. (a) Fluorescence intensity at 485 nm of DSH in different human serum fractions upon addition of HOCl at 0, 5, and 10 equiv. relative to DSH. (b) Fluorescence emission spectra of DSH in 3% human serum upon addition of increasing amounts of HOCl. (c) Calibration plot of fluorescence intensity at 485 nm *versus* HOCl equiv. in 3% human serum. Measurements were performed under the general fluorescence conditions, except that HSA was replaced with diluted human serum.

To evaluate the feasibility of DSH under diluted human serum conditions, the fluorescence response was examined in diluted human serum. First, the serum fraction was varied from 1% to 10%, and HOCl was added at 0, 5, or 10 equiv. relative to DSH. As shown in Fig. 6a, the fluorescence intensity increased with increasing serum fraction. This trend was likely due to the greater amount of serum albumin available for binding to DS released after the HOCl-mediated cleavage of DSH. A serum fraction-dependent background signal was also observed in the absence of HOCl, probably due to nonspecific interactions in the protein-rich serum matrix. Nevertheless, clear HOCl-dependent fluorescence enhancement was preserved at all the tested serum fractions, indicating that DSH can respond to HOCl in diluted human serum.

For calibration-based quantitative detection, 3% human serum was selected because its estimated albumin concentration is comparable to the optimised HSA concentration used in the PBS/HSA assay. Human serum albumin is present in serum at approximately 35–50 g L^−1^, corresponding to 0.53–0.75 mM based on its molecular weight.^35^ Therefore, 3% human serum is expected to contain approximately 16–23 µM albumin. Under this condition, increasing concentrations of HOCl induced a gradual fluorescence enhancement at the emission wavelength corresponding to the DS-albumin complex (Fig. 6b). The fluorescence intensity at 485 nm showed a linear relationship with the HOCl concentration over the tested range, allowing construction of a calibration curve for 3% human serum (Fig. 6c). The detection limit was calculated to be 0.54 µM using the 3*σ*/*k* method. These results indicated that DSH enables calibration-based fluorescence detection of HOCl in diluted human serum, supporting the feasibility of the albumin-assisted caged-fluorophore strategy for calibration-based detection in diluted serum.

To further evaluate the quantitative performance of DSH in the serum matrix, spike-recovery analysis was performed in 3% and 10% human serum. HOCl was spiked at final concentrations of 10 and 50 µM. Recovery values were calculated using separate matrix-matched calibration curves established over the 0–10 equiv. HOCl range for each serum fraction. In 3% human serum, recoveries of 95.52 and 98.83% were obtained for 10 and 50 µM HOCl, respectively, with RSD values of 2.43 and 0.57% (*n* = 3). In 10% human serum, the corresponding recoveries were 102.98 and 101.38%, with RSD values of 1.30 and 1.15% (*n* = 3) (Table 1). Although the baseline fluorescence varied with serum fraction, acceptable recovery and repeatability were obtained over the tested concentration range when matrix-matched calibration was used. These results suggest that serum-dependent matrix effects can be reasonably accommodated under the tested diluted-serum conditions by using matrix-matched calibration.

**Table 1:** Spike-recovery analysis of HOCl in 3% and 10% human serum using DSH. Matrix-matched calibration equations used for the recovery calculation: 3% serum, *F*_485_ = 11.17029*x* + 49.62675 (*R*^2^ = 0.9946); 10% serum, *F*_485_ = 5.57421*x* + 137.069534(*R*^2^ = 0.9858), where *F*_485_ is the fluorescence intensity at 485 nm and *x* is the HOCl concentration expressed as equivalents relative to DSH (10 µM)

Human serum (%)	Spiked HOCl (µM)	Found HOCl (µM)	Average recovery (%, *n* = 3)	RSD (%, *n* = 3)
3	0	ND	—	—
	10	9.55	95.52	2.43
	50	49.41	98.83	0.57
10	0	ND	—	—
	10	10.30	102.98	1.30
	50	50.69	101.38	1.15

## Conclusions

We developed DSH as an HSA-assisted caged fluorophore probe for the fluorescence turn-on detection of HOCl. DSH showed a clear HOCl-dependent fluorescence response under the optimised PBS/HSA conditions, enabling calibration-based detection with a detection limit of 0.66 µM. The sensing system exhibited selective fluorescence enhancement toward HOCl over other reactive oxygen and nitrogen species, and the HOCl-dependent response was preserved in the presence of the tested metal ions. LC-TQMS analysis supported the HOCl-mediated conversion of DSH to the albumin-binding fluorophore DS. Furthermore, DSH enabled calibration-based detection of HOCl in diluted human serum, and spike-recovery analysis showed acceptable recovery and repeatability in both 3% and 10% serum when matrix-matched calibration was employed. Taken together, these results support the feasibility of the albumin-assisted caged-fluorophore strategy for HOCl detection under defined PBS/HSA and diluted human serum conditions.

## Author contributions

Hyun Seo Kim: methodology, investigation, visualisation, writing – original draft. Min Su Han: conceptualisation, methodology, supervision, project administration, funding acquisition, resources, writing – review & editing.

## Conflicts of interest

There are no conflicts to declare.

## Supplementary Material

RA-OLF-D6RA06489B-s001

## Data Availability

The data supporting this article have been included as part of the supplementary information (SI). Supplementary information: synthetic procedures, characterisation data, and supplementary fluorescence measurements. See DOI: https://doi.org/10.1039/d6ra06489b.
