# Supplementary material for: A human serum albumin-assisted caged fluorophore probe for fluorescence turn-on detection of hypochlorous acid
Source: RSC Adv. 2026 Sep 18;16(44):55703–9. doi: 10.1039/d6ra06489b (PMC13586984; doi:10.1039/d6ra06489b)
Supplement: Supplementary file 1 [file RA-016-D6RA06489B-s001.pdf]

Supplementary Information (SI) for RSC Advances.
This journal is © The Royal Society of Chemistry 2026

Electronic Supplementary Information (ESI)

A Human Serum Albumin-Assisted Caged Fluorophore Probe for Fluorescence Turn-On Detection of Hypochlorous Acid

Hyun Seo Kim and Min Su Han*

Department of Chemistry, Gwangju Institute of Science and Technology, 123 Cheomdangwagi-ro, Buk-gu, Gwangju 61005, Republic of Korea

E-mail: happyhan@gist.ac.kr; Fax: +82-62-715-2866; Tel: +82-62-715-2848

Table of Contents

1. Experimental procedures..S1

2. Supplementary data S2

1. Experimental procedures

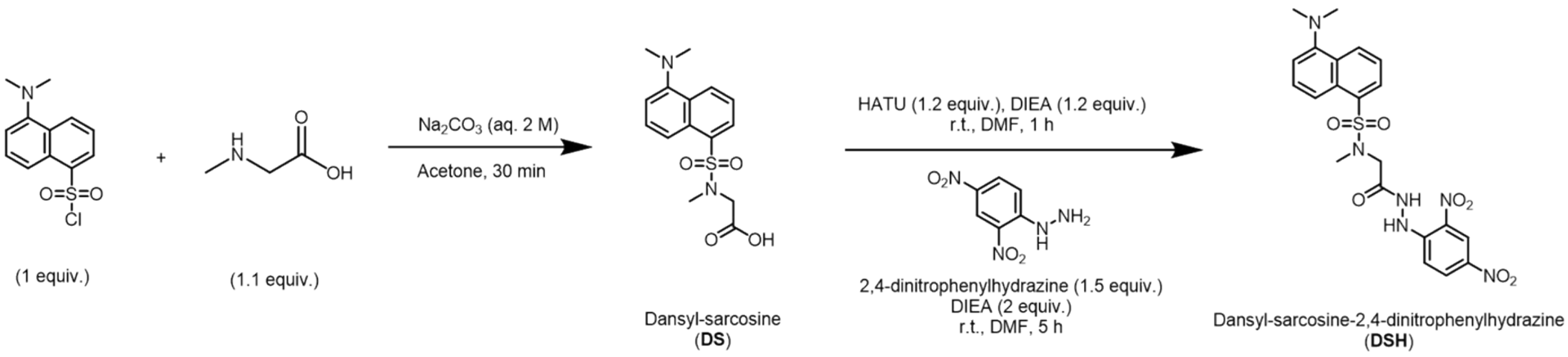

Figure S1. Reaction scheme for the synthesis of **DSH**.

Synthesis of Dansyl-sarcosine (DS)

Following a literature procedure,[1] dansyl chloride (0.270 g, 1.00 mmol) was dissolved in acetone and filtered to remove insoluble white solid impurities. Sarcosine (0.098 g, 1.10 mmol) was dissolved in aqueous Na_2CO_3 solution (2 M, 1.4 mL), and the resulting solution was added dropwise to the dansyl chloride solution using a syringe. The reaction mixture was stirred at room temperature for 30 min. The reaction mixture was monitored by TLC using dichloromethane/methanol (10:1, v/v), which showed the formation of a new fluorescent spot (R_f = 0.1). After completion of the reaction, dichloromethane was added, and the mixture was subjected to basic aqueous extraction using Na_2CO_3 solution to transfer the product into the aqueous phase. The aqueous layer was then acidified to pH 3 with aqueous HCl, and the product was extracted with dichloromethane. The combined organic layers were dried over anhydrous Na_2SO_4, filtered, and concentrated under reduced pressure to afford **DS** as a pale yellow solid in 76% yield. ^{1}H NMR (400 MHz, DMSO-d_6) δ 8.48 (d, J = 8.7 Hz, 1H), 8.16 (dd, J = 32.3, 8.0 Hz, 2H), 7.62 (q, J = 8.2 Hz, 2H), 7.26 (d, J = 7.8 Hz, 1H), 4.07 (s, 2H), 2.90 (s, 3H), 2.82 (s, 6H). ^{13}C NMR (101 MHz, DMSO-d_6) δ 170.1, 151.4, 134.9, 129.8, 129.3, 128.4, 123.7, 119.0, 115.3, 50.2, 45.1, 35.4.

2. Supplementary data

Verification of HOCl stock concentration

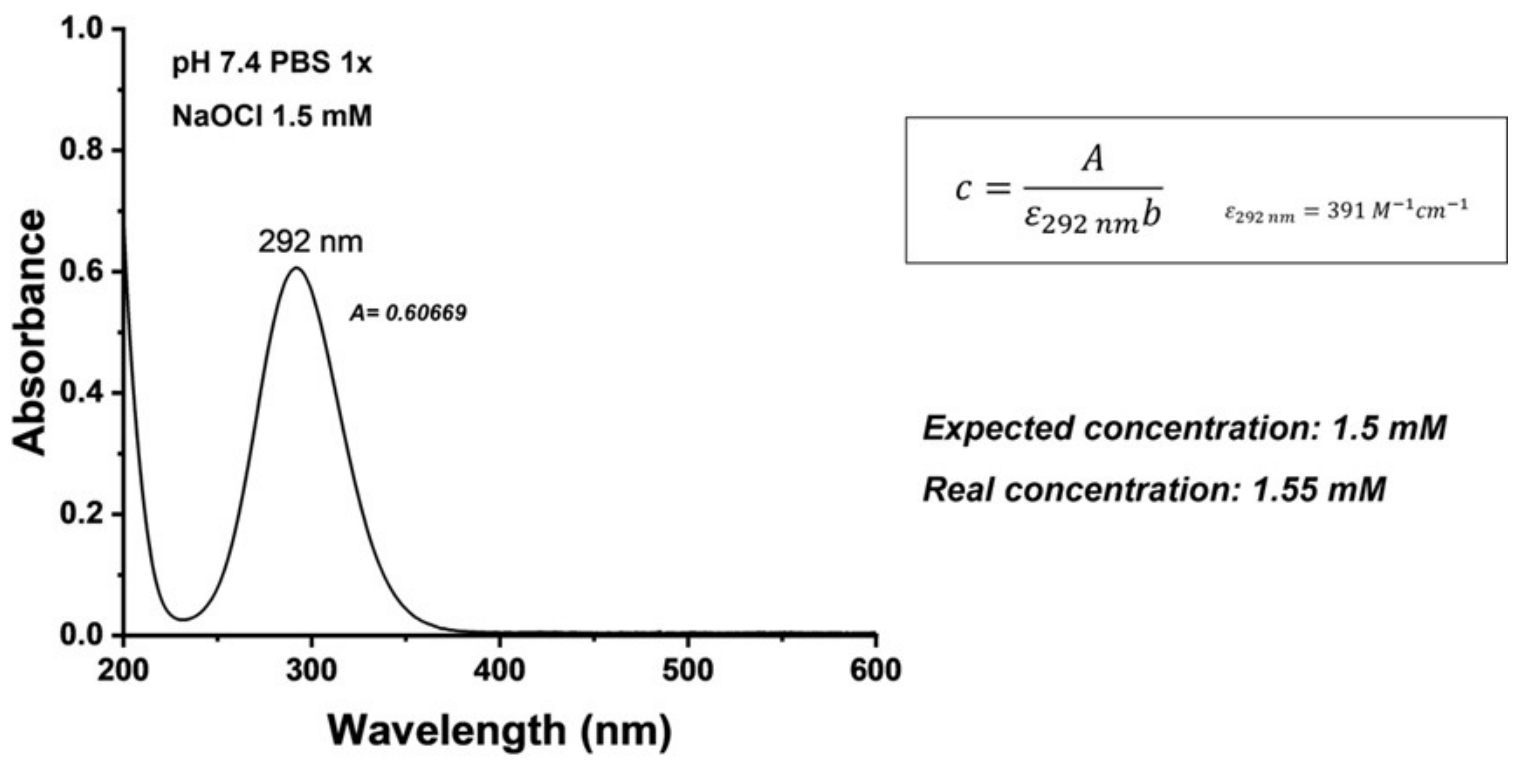

Figure S2. UV-Vis absorption spectrum used to verify the concentration of the HOCl stock solution. The HOCl concentration was calculated from the absorbance at 292 nm using the reported molar extinction coefficient.[2]

HSA-dependent fluorescence behaviour of DS

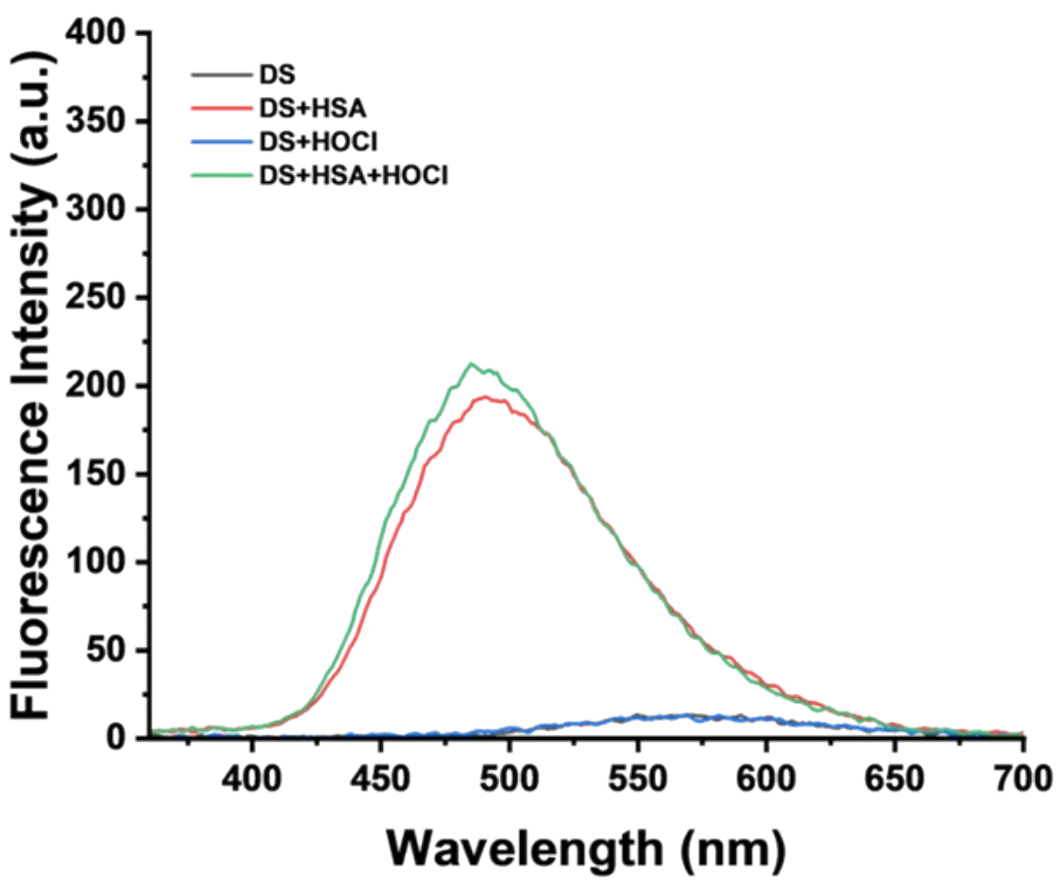

Figure S3. Fluorescence emission spectra of **DS** in the absence and presence of HSA, HOCl, or both. **DS** = 10 µM, HSA = 20 µM, HOCl = 100 µM, λ_{ex} = 340 nm, and slit widths = 5/5 nm. Measurements were performed after incubation for 90 min at room temperature.

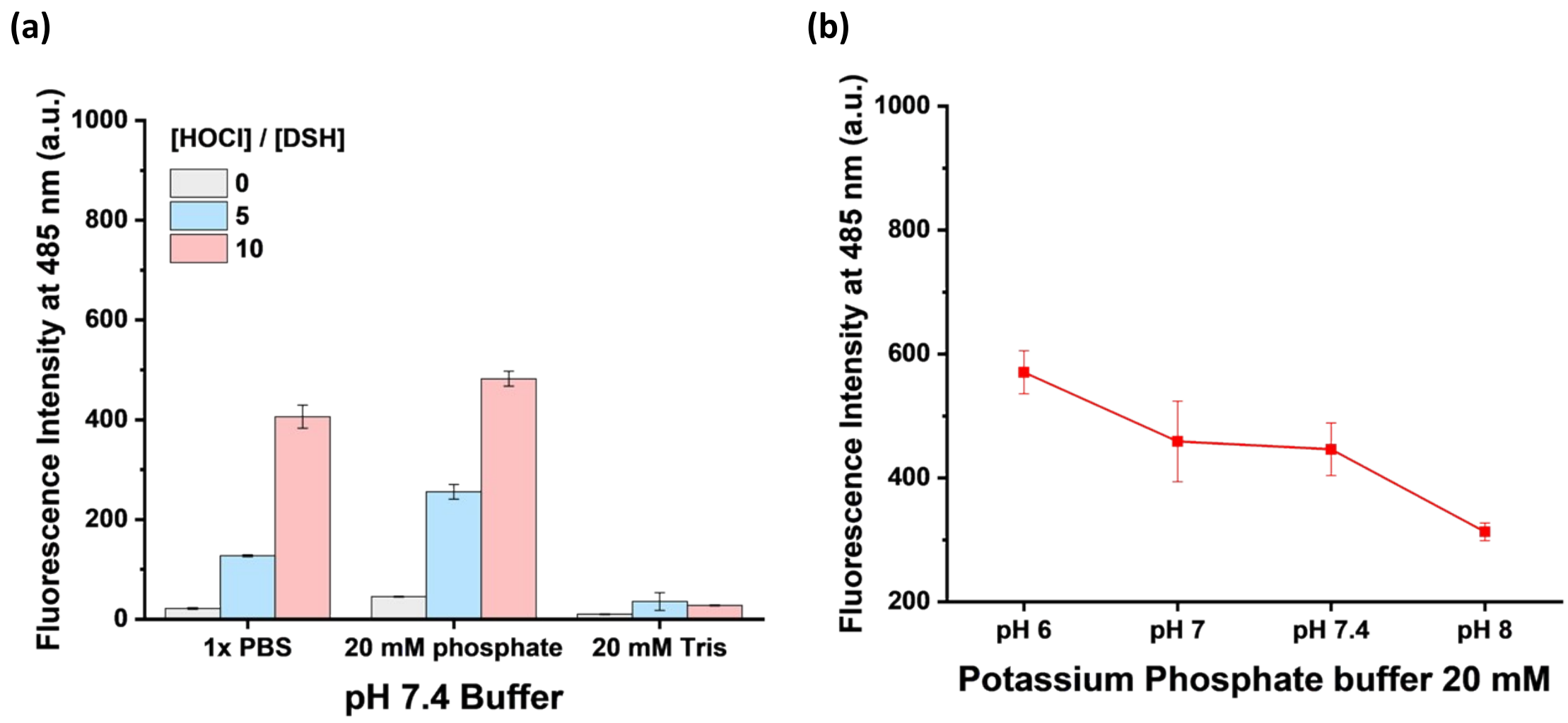

Buffer and pH effects on the fluorescence response of DSH

Figure S4. Effects of buffer and pH on the fluorescence response of **DSH** toward HOCl. (a) Fluorescence intensity at 485 nm of **DSH** in different buffer systems after the addition of HOCl at 0, 5, and 10 equiv. relative to **DSH**. (b) Fluorescence intensity at 485 nm of **DSH** toward 100 µM HOCl under different pH conditions. Unless otherwise stated, **DSH** = 10 µM, HSA = 20 µM, λ_{ex} = 340 nm, and measurements were performed after incubation for 90 min at room temperature.

HSA-dependent fluorescence response of DSH toward HOCl

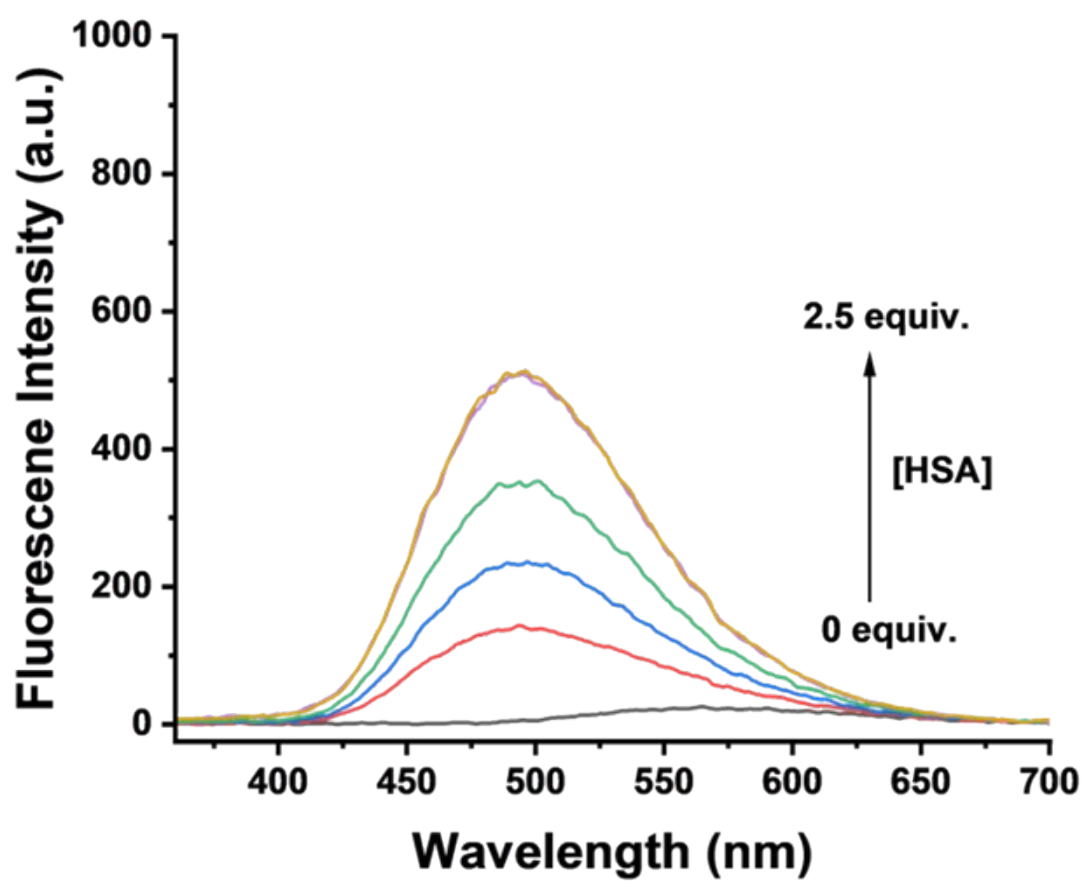

Figure S5. HSA-dependent fluorescence response of **DSH** toward HOCl. Fluorescence emission spectra of **DSH** recorded after treatment with HOCl in the presence of increasing HSA concentrations. **DSH** = 10 µM, HSA = 0–25 µM, HOCl = 100 µM, λ_{ex} = 340 nm, and measurements were performed in 1× PBS (pH 7.4) containing 1% DMSO after incubation for 90 min at room temperature.

(a)

(b)

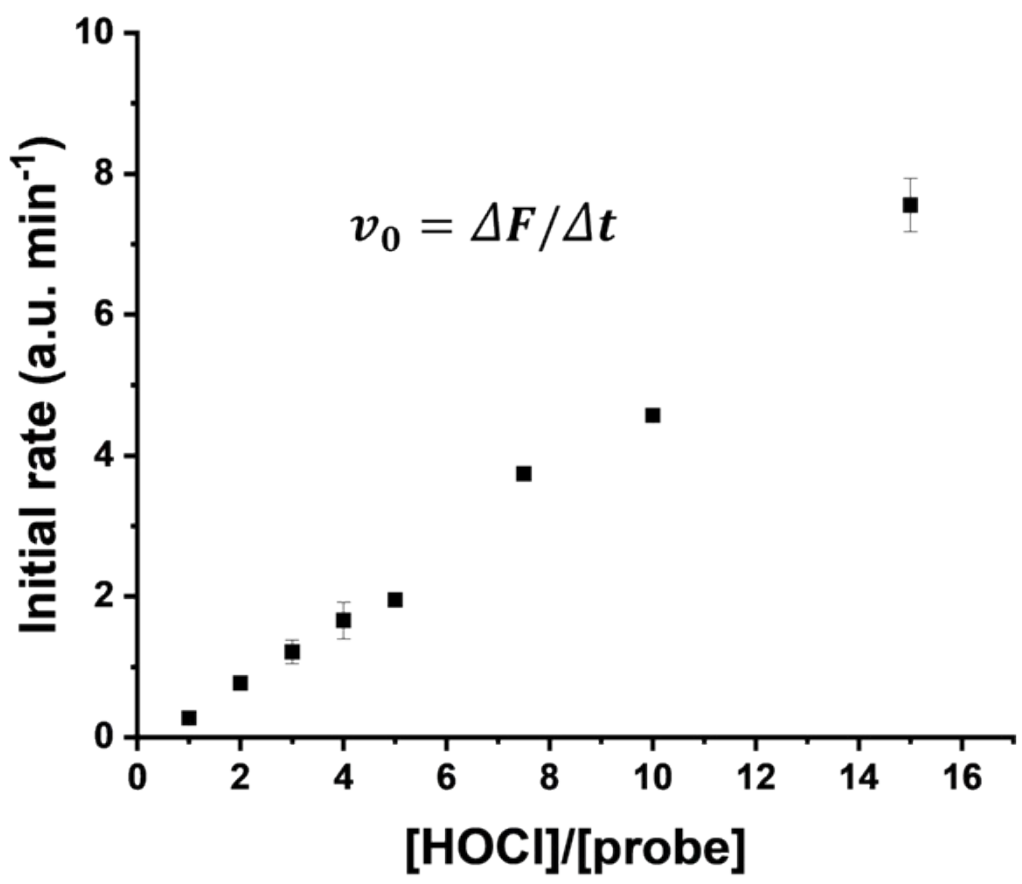

Kinetic analysis

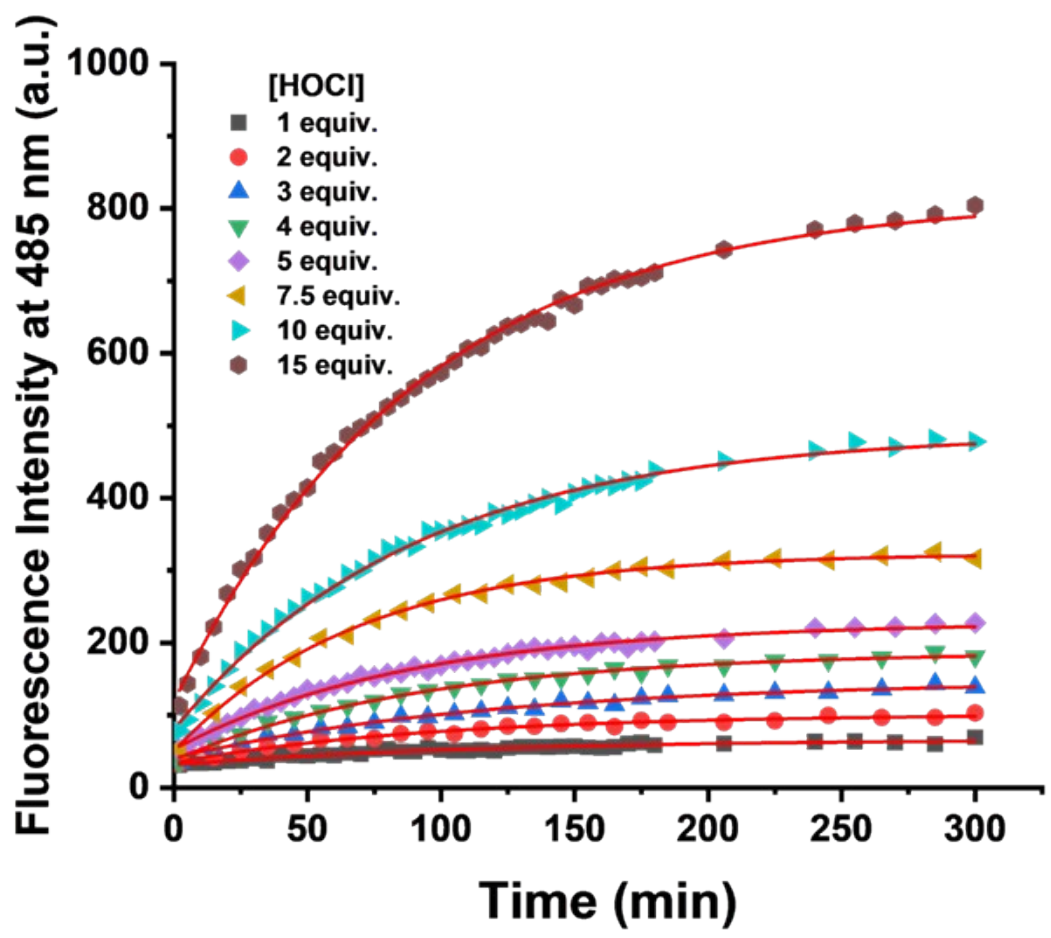

Figure S6. Kinetic analysis of the fluorescence response of **DSH** toward HOCl. (a) Time-dependent fluorescence response of **DSH** at different HOCl concentrations. (b) Apparent initial fluorescence increase rates ($v_0 = \Delta F/\Delta t$), determined from linear fits to the first 30 min of each time course, plotted as a function of HOCl equivalents. Unless otherwise stated, **DSH** = 10 µM, HSA = 20 µM, λ_{ex} = 340 nm, and fluorescence intensity was monitored at 485 nm.

Early-time calibration of DSH toward HOCl

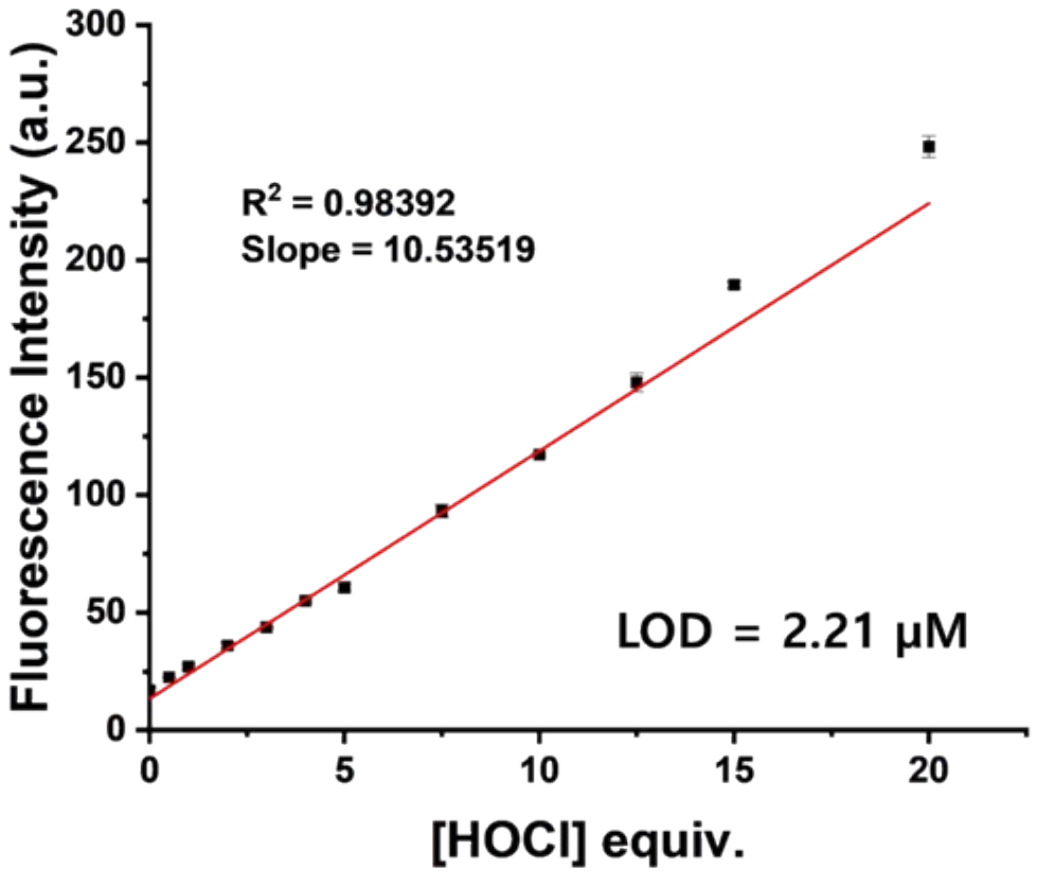

Figure S7. Calibration plot of fluorescence intensity at 485 nm versus HOCl equiv. after incubation for 10 min. **DSH** = 10 µM, HSA = 20 µM, λ_{ex} = 340 nm, and measurements were performed in 1× PBS (pH 7.4) containing 1% DMSO at room temperature.

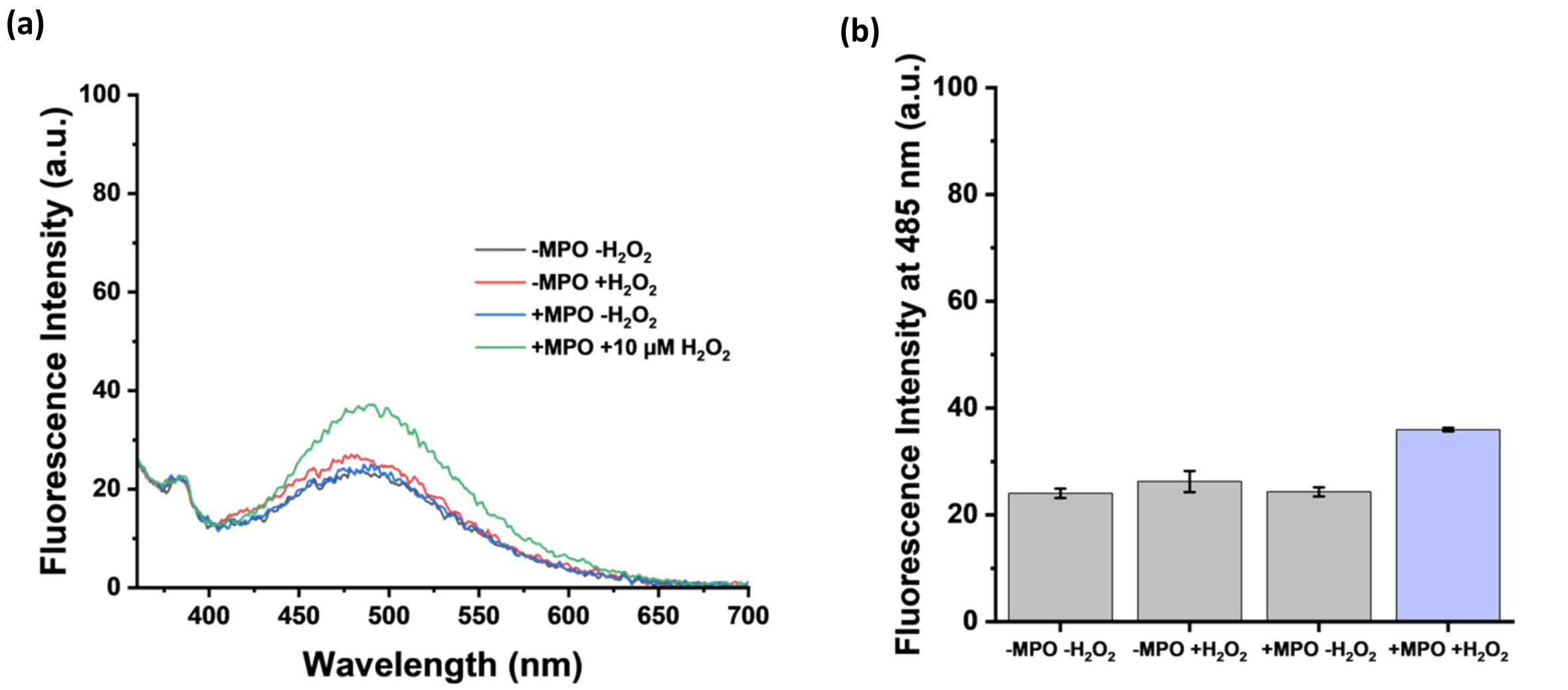

MPO/H_2O_2-mediated in situ HOCl-generation assay

Figure S8. Fluorescence response of **DSH** under an MPO/H_2O_2-mediated HOCl-generation condition. (a) Fluorescence emission spectra of **DSH** in the absence or presence of MPO and H_2O_2. (b) Corresponding fluorescence intensities at 485 nm. **DSH** = 10 µM, HSA = 20 µM, MPO = 0.07 U/mL, H_2O_2 = 10 µM, and λ_{ex} = 340 nm. Measurements were performed in 1× PBS (pH 7.4) containing 1% DMSO after incubation for 90 min at room temperature.

NMR spectra

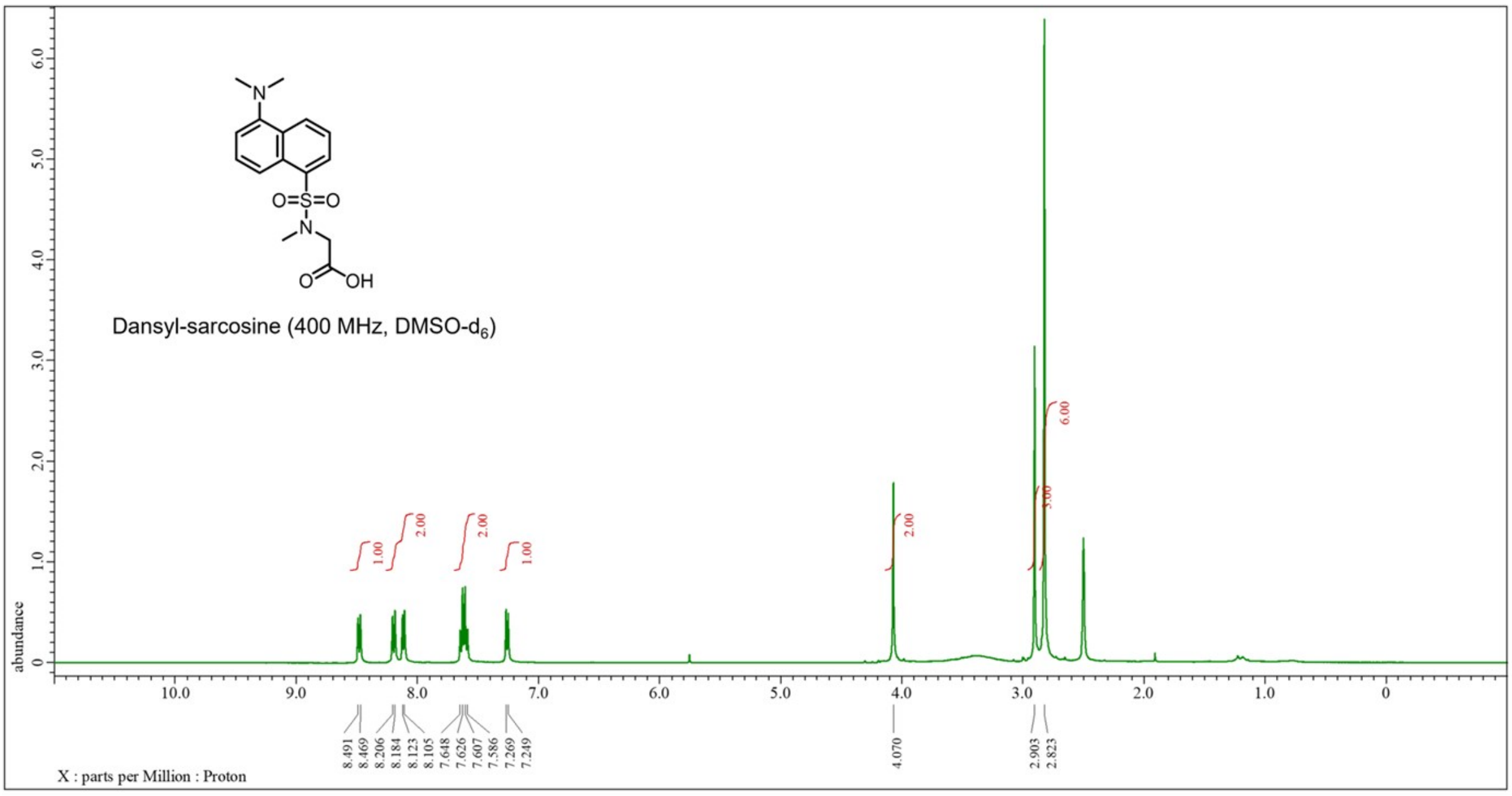

Figure S9. ^{1}H NMR spectrum of **DS**.

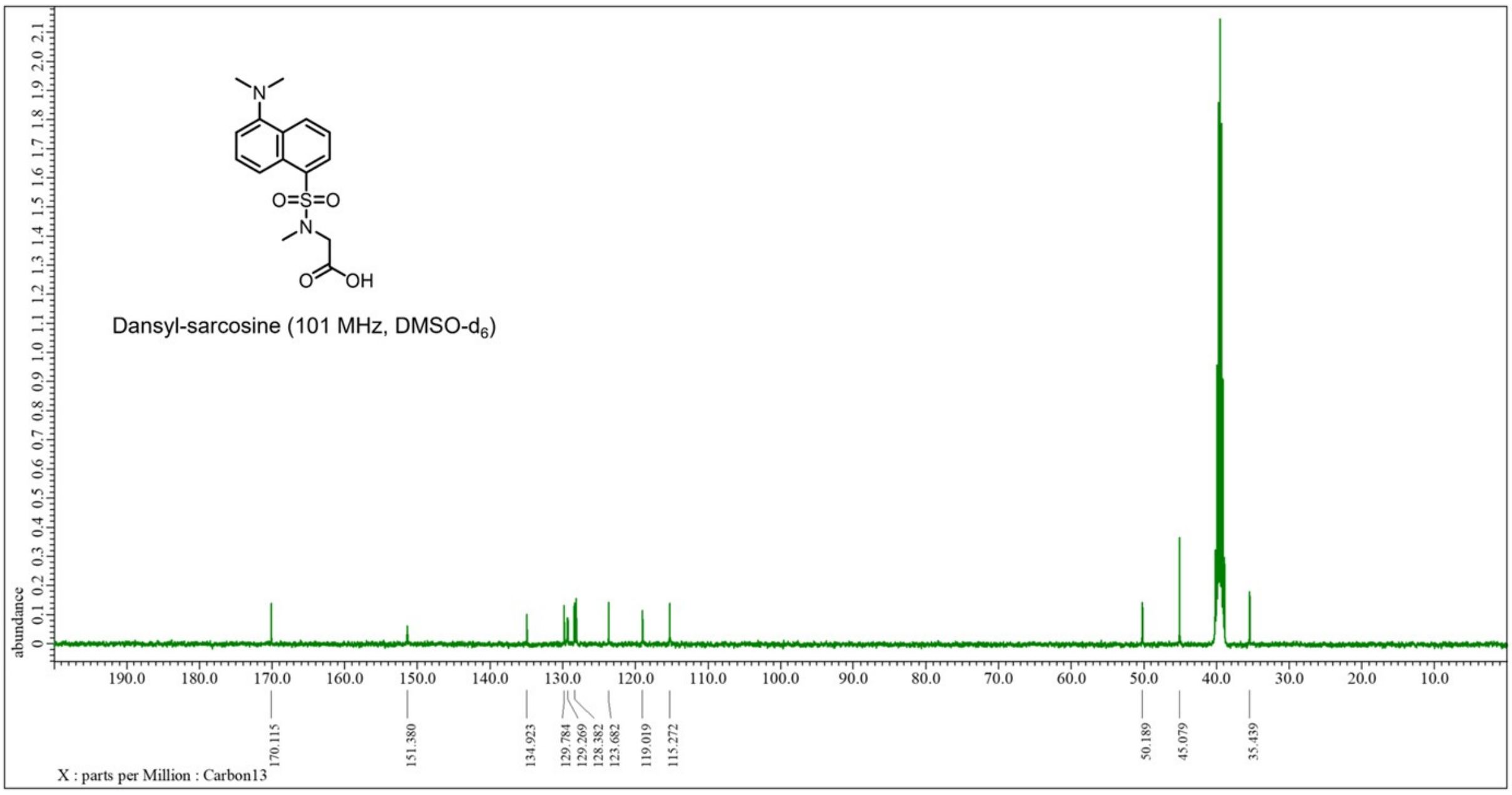

Figure S10. ^{13}C NMR spectrum of **DS**.

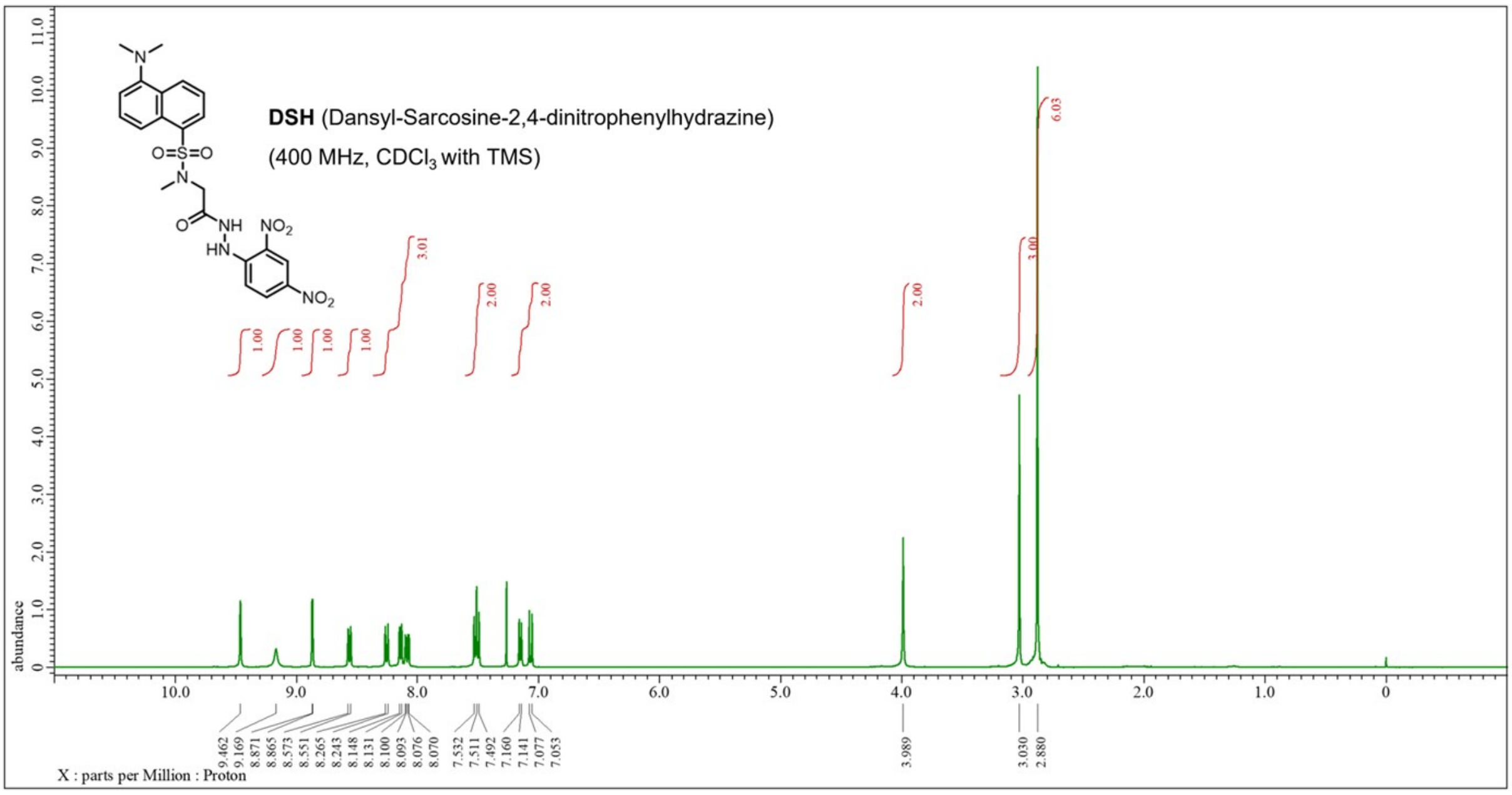

Figure S11. ^{1}H NMR spectrum of **DSH**.

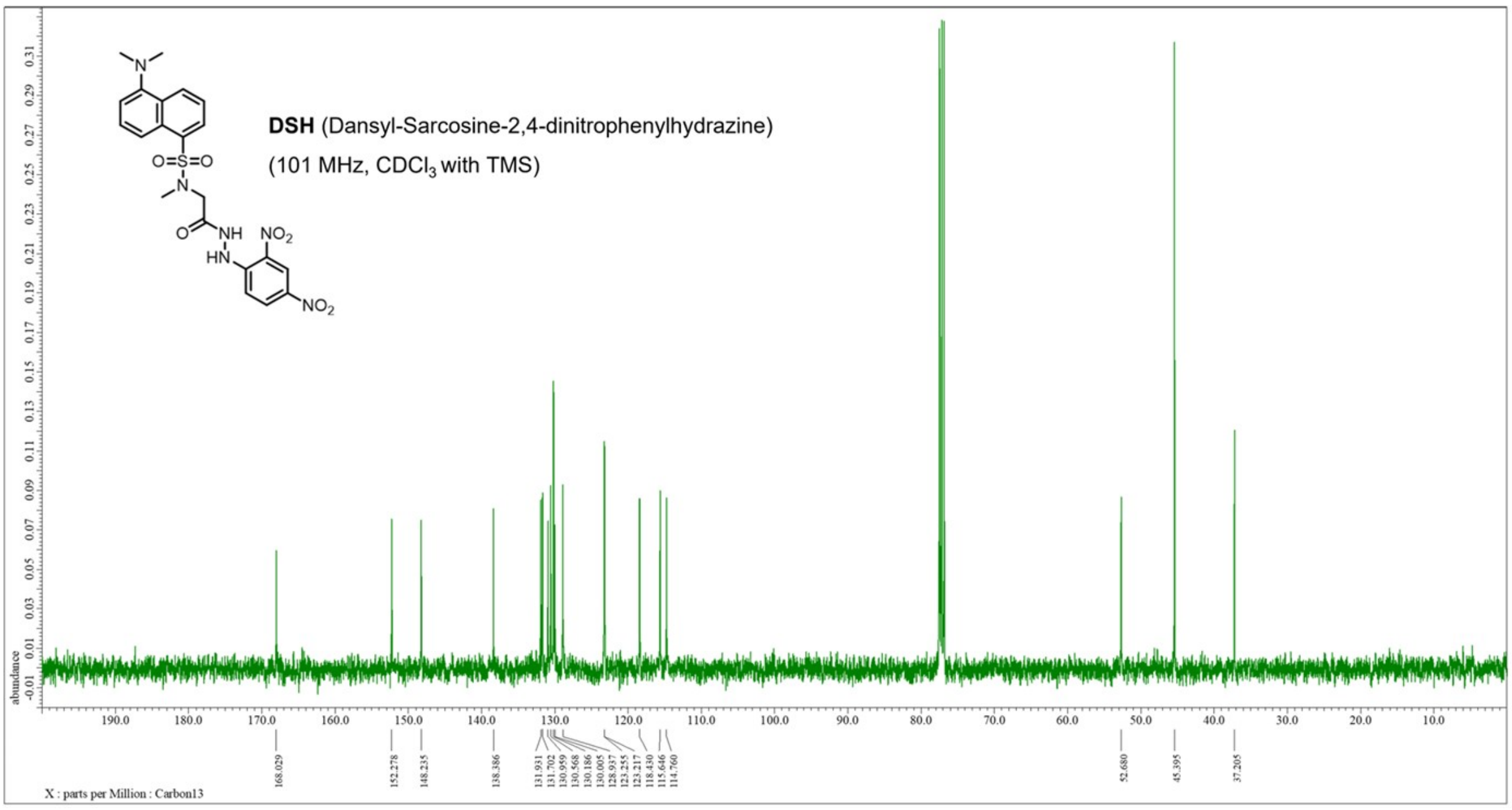

Figure S12. ^{13}C NMR spectrum of **DSH**.

(a)

(b)

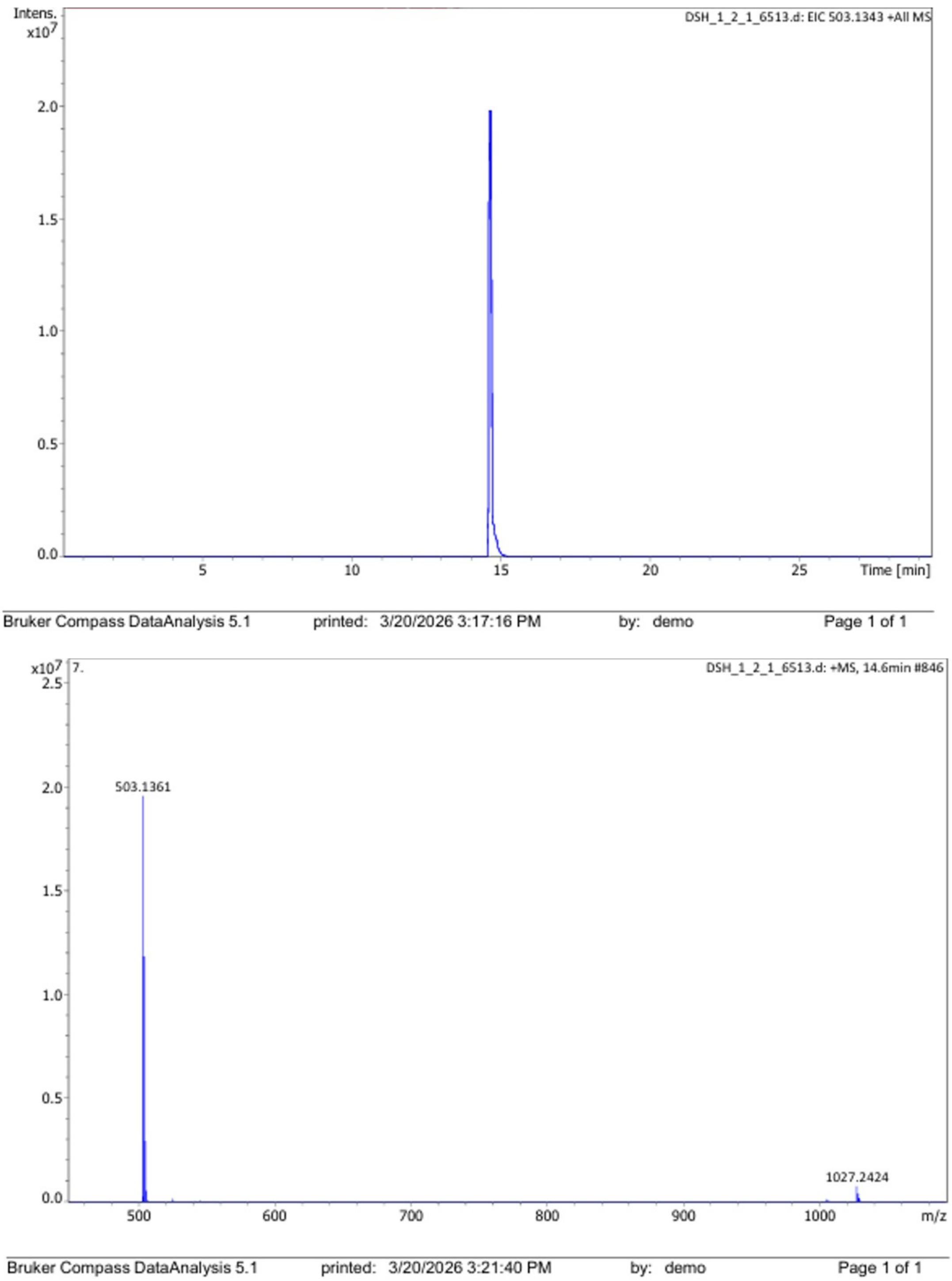

High-resolution mass spectra

Figure S13. LC-QTof-MS analysis of **DSH**: (a) extracted ion chromatogram and (b) mass spectrum.

Probe	Journal, year	Protein-assisted signal generation	Serum/plasma validation	**LOD (nM)**	Reported response time	Ref.
**HKOCl-3**	*Chem. Sci.*, 2016	None	Not evaluated	0.33	Within 1 min	3
**TPM**	*J. Mater. Chem. B*, 2019	BSA-assisted	Not evaluated	1.5	Within seconds	4
**HBI-based probe**	*RSC Adv.*, 2019	None	Human serum; protein precipitation + 50-fold dilution; spike/recovery	14.6	30 s	5
**BNA-HClO**	*RSC Adv.*, 2022	None	Fetal bovine serum; spike/recovery	37.56	<30 s	6
**TPBN**	*Org. Biomol. Chem.*, 2024	None	Not evaluated	8.74	80 s	7
**PTZP**	*J. Photochem. Photobiol., A*, 2026	None	Not evaluated	40	6 s	8
**IPH1**	*Spectrochim. Acta, Part A*, 2026	None	Not evaluated	29	$t_{1/2}$ = 15 s	9
**HClO-CyOH-S**	*Spectrochim. Acta, Part A*, 2026	None	Blood plasma; sepsis diagnosis	200	Within 3 min	10
**DSH**	This work	HSA-assisted	Diluted human serum; calibration-based detection at 3%; spike/recovery at 3% and 10%; no protein removal	660 (PBS/HSA); 540 (3% serum)	Response apparent at 10 min; 90 min endpoint	This work

Table S1. Comparison of representative fluorescent probes for HOCl detection.

References

1. S. Yoo and M. S. Han, *Chem. Commun*., 2019, **55**, 14574.
2. S. Chen, J. Lu, C. Sun and H. Ma, *Analyst*, 2010, **135**, 577.
3. J. J. Hu, N. Wong, M. Lu, X. Chen, S. Ye, A. Q. Zhao, P. Gao, R. Yi-Tsun Kao, J. Shen and D. Yang, *Chem. Sci.*, 2016, **7**, 2094.
4. P. Zhang, Q. Zhang, S. Li, W. Chen, X. Guo and C. Ding, *J. Mater. Chem. B*, 2019, **7**, 1238.
5. J. Li, A. Gong, G. Shi and C. Chai, *RSC Adv.*, 2019, **9**, 30943.
6. W. Hu, M. Zhao, K. Gu, L. Xie, M. Liu and D. Lu, *RSC Adv.*, 2022, **12**, 777.
7. S. Banerjee, D. Banik, S. Halder, A. Karak, P. Ghosh, K. Jana and A. K. Mahapatra, *Org. Biomol. Chem.*, 2024, **22**, 1662.
8. X. Tang, J. Fan, J. Liao, S. Tang and Y. Han, *J. Photochem. Photobiol., A*, 2026, **473**, 116922.
9. S. Li, J. Fan, R. Yang and Y. Han, *Spectrochim. Acta, Part A*, 2026, **348**, 127216.
10. Q.-Q. Wang, X.-Y. Sun, S.-Z. Liu, T.-T. Yang, R. Rong, T.-F. Chen, H. Wang and G.-J. Mao, *Spectrochim. Acta, Part A*, 2026, **354**, 127620.
